# Advancing Sleep Testing for Children: In-Laboratory Validation of a Type 2 Sleep-Monitoring System Designed for Home-Based Pediatric Assessment

**DOI:** 10.3390/children13101432

**Published:** 2026-10-18

**Authors:** Benjamin D. Fox, Murad Shihab, Dahlia Kushinsky, Saar Lanir-Azaria, Abed Nassir, Ofer Barnea, Asher Tal

**Affiliations:** 1 Pulmonary Division, Shamir Medical Center, Be’er Ya’akov 7033001, Israel; 2 Sackler Faculty of Medicine, Tel Aviv University, Tel Aviv 6997801, Israel; 3 Dormotech Medical, Dolev 4, Ra’anana 4366204, Israelabed@dormotech.com (A.N.); 4 Department of Biomedical Engineering, Tel Aviv University, Tel Aviv 6997801, Israel; 5 Department of Medicine, Faculty of Health Sciences, Ben-Gurion University of the Negev, Beer-Sheva 8410501, Israel

**Keywords:** home sleep study test, pediatric sleep study, wearable monitoring, sleep-disordered breathing, apnea–hypopnea index

## Abstract

**Highlights:**

**What are the main findings?**

• Simultaneous recordings showed strong agreement between the investigational Dormotech Type 2 sleep monitoring system and in-laboratory polysomnography for respiratory indices and sleep-wake classification in children and adolescents.

**What are the implications of the main findings?**

• Comprehensive Type 2 sleep monitoring can capture both respiratory and neurophysiological sleep measures in pediatric patients, addressing current limitations in pediatric sleep monitoring technologies.
• These findings support the advancement of comprehensive sleep testing as an accessible, patient-centered approach to pediatric sleep assessment, with the potential to expand diagnostic capabilities beyond specialized sleep laboratories.

**Abstract:**

Background: Comprehensive Type 2 sleep-monitoring systems designed to support home-based assessment may improve access to pediatric sleep assessment while preserving evaluation of sleep architecture and respiratory events. This study evaluated the clinical performance of a wearable Type 2 sleep monitoring system compared with standard in-laboratory polysomnography in children. Methods: Children referred for overnight sleep evaluation underwent simultaneous recordings using the Dormotech wearable Type 2 system and in-laboratory polysomnography (Nox A1). Recordings were manually scored according to American Academy of Sleep Medicine guidelines. Primary outcomes were apnea-hypopnea index (AHI) and severity classification; secondary outcomes included global sleep-wake and respiratory parameters, usability, and safety. Results: Twenty-four children completed supervised in-laboratory recordings and were included in the analysis (median age, 10.5 years; 25th–3.6) events/h with polysomnography (*p* = 0.808). AHI severity classification showed high agreement (κ = 0.896), with a med75th percentiles, 8.8–13.3; 50.0% female). Median AHI was 2.2 (25th–75th percentiles, 0.9–3.4) events/h with Dormotech and 1.3 (0.6–3.6) events/h with polysomnography (*p* = 0.808). AHI severity classification showed high agreement (κ = 0.896), with a median Bland—Altman paired difference (Nox − Dormotech) of −0.2 events/h and empirical 95% limits of agreement of −1.9 to 6.7 events/h. No significant differences were observed across secondary sleep or respiratory parameters. User experience was favorable, with no device-related adverse events. Conclusions: The comprehensive Type 2 system demonstrated strong agreement with in-laboratory polysomnography across respiratory and global sleep-wake measures in children. These findings demonstrate agreement under supervised laboratory conditions and provide a foundation for future studies evaluating the system’s performance in unsupervised use settings.

## 1. Introduction

Sleep disorders are common in children and can substantially impair neurocognitive development, behavior, cardiovascular health, growth, and quality of life [1,2,3]. Sleep-disordered breathing (SDB), particularly obstructive sleep apnea (OSA), is among the most prevalent pediatric sleep disorders, affecting approximately 1–3% of preschool- and school-aged children, and has been associated with impaired attention, learning, emotional regulation, and long-term cardiometabolic consequences when left untreated [1,2,4]. Early diagnosis is therefore essential to enable timely intervention during critical stages of development.

Despite this clinical need, access to pediatric polysomnography (PSG) remains limited worldwide because of the scarcity of specialized pediatric sleep laboratories, long waiting lists, and the logistical burden imposed on children and their families [5,6]. Overnight laboratory studies also require extensive instrumentation in an unfamiliar environment, which may increase anxiety, reduce comfort, and influence habitual sleep patterns, particularly in younger children [6,7]. Consequently, there is growing interest in ambulatory sleep-monitoring approaches that can be used outside specialized sleep laboratories, including systems that may be applied independently by children and their caregivers, for pediatric sleep assessment while maintaining the diagnostic accuracy required for clinical decision-making [3].

Pediatric sleep assessment, however, presents distinct diagnostic challenges compared with adult sleep medicine. OSA is diagnosed at substantially lower AHI thresholds in children, such that relatively small differences in respiratory event detection may influence diagnostic classification. Moreover, accurate characterization of pediatric SDB frequently depends on reliable identification of sleep state and cortical arousals in accordance with American Academy of Sleep Medicine (AASM) criteria [4,8]. Comprehensive physiological monitoring, including electroencephalography (EEG), electrooculography (EOG), electromyography (EMG), and respiratory signals, therefore remains fundamental to pediatric sleep evaluation. Nevertheless, most currently available home sleep testing (HST) systems were developed primarily for adults and focus predominantly on respiratory measurements, limiting their ability to characterize sleep architecture and other polysomnographic features relevant to pediatric assessment [5,6].

Comprehensive Type 2 ambulatory sleep-monitoring systems capable of HST have the potential to bridge this gap by combining the physiological information traditionally obtained during laboratory PSG with the accessibility of home-based sleep assessment. If validated for unattended home use, such systems may enable assessment in the child’s natural sleep environment while preserving the physiological information required for comprehensive pediatric sleep evaluation. In the present study, we evaluated a wearable Type 2 ambulatory sleep-monitoring system capable of HST recording designed for pediatric use. To isolate differences attributable to the recording systems rather than the recording environment, simultaneous recordings were performed under supervised laboratory conditions alongside standard in-laboratory PSG to assess agreement in respiratory events, global sleep–wake measures, and other clinically relevant sleep parameters, as well as device usability and safety.

## 2. Materials and Methods

### 2.1. Study Design

This prospective, self-controlled study was conducted from 5 January 2024 to 11 January 2025 and was approved by the institutional review board of Shamir Medical Center (approval number 0067-23-ASF). The studywas conducted in accordance with the Declaration of Helsinki and applicable regulatory guidelines. Parental and participant informed consent were obtained prior to study participation.

This study was conducted at Shamir Medical Center Sleep Laboratory (Be’er Ya’akov, Israel) and Millennium Sleep Clinic (Be’er Sheva, Israel). Children aged ≥6 years referred for diagnostic PSG were eligible. The exclusion criteria were as follows: (i) known allergies to the device materials, (ii) irritated skin, (iii) open wounds on the body at the site of device placement, or (iv) implanted or external electronic devices of any kind. All participants were enrolled without investigator pre-selection. The study was designed to evaluate overall agreement between the investigational system and reference polysomnography across the pediatric cohort and was not powered to perform age-stratified subgroup analyses. Participants underwent simultaneous overnight recordings using the investigational Dormotech system and the reference standard Nox A1 (Noxturnal Software System, version 6.3) PSG system routinely used in the sleep laboratory. Usability was assessed using pre- and post-procedure questionnaires. The scored data from both devices were analyzed to determine agreement in the calculated parameters between the two devices.

### 2.2. Overnight Polysomnography

**Figure 1:**
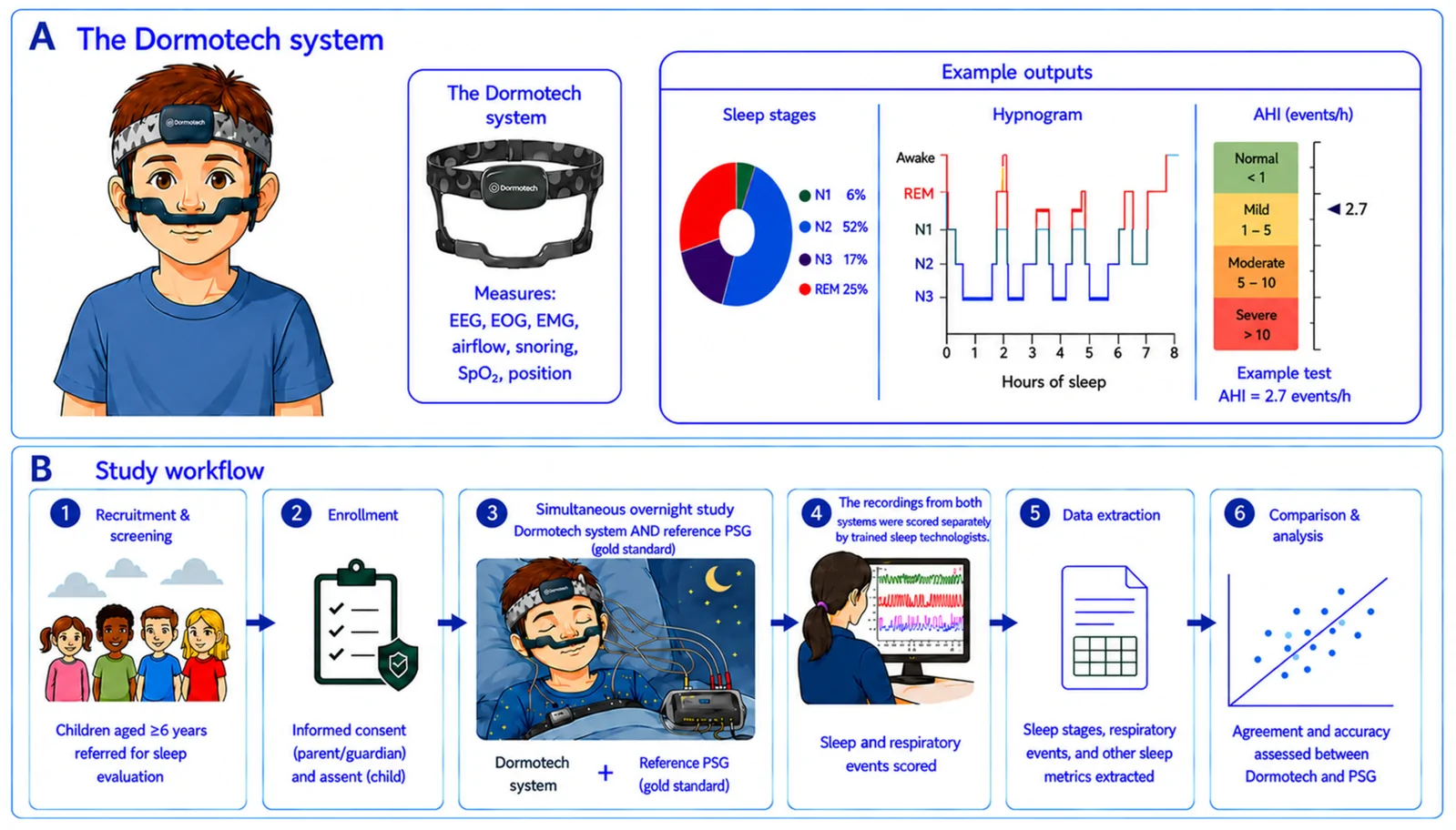
Overview of the Dormotech system and study workflow. (**A**) Schematic overview of the investigational Dormotech system used in the study. The Dormotech system records electroencephalography (EEG), electrooculography (EOG), electromyography (EMG), nasal and oral airflow, snoring, ECG, thoracic and abdominal respiratory effort, and head and body position. Peripheral oxygen saturation (SpO_2_) and pulse rate are obtained from a separate photoplethysmography-based pulse-oximetry sensor. Representative system outputs include sleep stage distribution, a hypnogram, and apnea–hypopnea index (AHI) severity classification. (**B**) Study workflow. Children referred for diagnostic sleep evaluation underwent simultaneous overnight recording with the investigational Dormotech system and the reference polysomnography (PSG) system during the same night. Recordings from the two systems were scored separately by trained sleep technologists, followed by extraction of sleep and respiratory parameters and statistical comparison between the two systems.

Overnight PSG was conducted using both the Dormotech device (Vlab; Dormotech Medical, Ra’anana, Israel) and the reference standard Nox A1 system (Nox Medical, Reykjavik, Iceland). Dormotech consisted of a wireless wearable head and torso unit that transmitted real-time data to a secure server via Wi-Fi (Figure 1). All recordings were performed in controlled laboratory environments at either center. Both systems were operated concurrently and captured physiological signals during the same sleep session. The Nox A1 system was used according to the standard clinical protocol and recorded multiple channels, including EEG, EOG, EMG, electrocardiogram, respiratory effort, airflow, snoring, and pulse oximetry. The Dormotech system comprised wearable head and torso units. The head unit recorded EEG, EOG, EMG, nasal and oral airflow, snoring, ECG, and accelerometers to assess head position. The torso unit included thoracic and abdominal belts for respiratory-effort measurement, with accelerometers used for body-position assessment. Peripheral oxygen saturation (SpO_2_) and pulse rate were obtained from a photoplethysmography (PPG) sensor. End-tidal CO_2_ data were not included in the study dataset. The Dormotech and Nox A1 systems used separate sensor sets and independently acquired their respective signals; no physical sensors were shared between the systems. No modifications were made to the clinical routine of either device.

### 2.3. Sleep Scoring

Raw data files were exported in European Data Format for manual scoring. Sleep staging and respiratory events were annotated using Noxturnal Software (version 6.3) in accordance with the American Academy of Sleep Medicine guidelines [8]. Apneas were defined as a ≥90% reduction in airflow lasting for at least two breaths. Hypopneas were defined as a ≥30% reduction in airflow lasting for at least two breaths and associated with either a ≥3% oxygen desaturation or an arousal. Trained technologists performed the scoring without access to participant identity. The same scorer evaluated both the Dormotech and Nox files for each participant to minimize inter-rater variability. To minimize the potential for recall bias, data from each device were scored in separate sessions separated by a 3–5-day washout period, with the Dormotech recordings scored without synchronized video.

### 2.4. Outcome Measures

The primary outcome measure was the apnea–hypopnea index (AHI), expressed as events per hour of sleep and categorized by severity (normal: <1, mild: 1–4.9, moderate: 5–9.9, severe: ≥10) [9]. The secondary outcome measures included total sleep time (TST), total recording time (TRT), sleep efficiency (% calculated as TST/TRT × 100), Wake percentage (calculated relative to TRT), sleep latency, and wake after sleep onset (WASO). Additional measures included oxygen desaturation index (ODI; events per hour), total snoring duration (as a percentage of TST), and body position (supine, left, right, or prone, expressed as a percentage of TST).

Device usability was assessed using a 5-point Likert-style scale questionnaire administered pre- and post-study (Supplementary Table S3). The participants, with the help of their caregivers, were instructed to evaluate only the Dormotech device. An acceptable usability threshold was set a priori at ≥70% of responses scoring 3 (neutral) or higher. Device safety was monitored via adverse event reporting.

### 2.5. Statistical Analysis

The target sample size was informed in part by the Food and Drug Administration (FDA) guidance Applying Human Factors and Usability Engineering to Medical Devices, which recommends including at least 15 participants from each distinct user population in usability validation studies. This recommendation informed the usability component of the study, while the clinical component was designed to evaluate agreement between the investigational system and reference PSG. Previous adult validation of the Dormotech platform further supported its evaluation in children [10]. Accordingly, 26 participants were recruited across the two study sites, of whom 24 completed simultaneous recordings and were included in the final analysis. Age, sex, and body mass index (BMI) were collected to characterize the cohort.

Categorical variables were summarized as frequencies and percentages, and continuous and ordinal variables as medians (25th–75th percentiles). Usability responses were additionally summarized as percentages of ratings ≥ 3. Normality of continuous baseline variables and paired differences (Nox − Dormotech) was assessed using Shapiro–Wilk tests. Two-sided paired *t*-tests were used when normality of paired differences was not rejected (*p* ≥ 0.05); otherwise, Wilcoxon signed-rank tests were applied using a tie-adjusted normal approximation, excluding zero differences and without continuity correction. AHI was the prespecified primary outcome, with no multiplicity adjustment; *p* values for the 11 secondary outcomes were jointly adjusted using the Benjamini–Hochberg false-discovery-rate procedure. Analyses used available valid pairs without imputation. *p* values were reported to three decimal places. Continuous-measure agreement was assessed using root mean square error (RMSE) and Bland–Altman analysis. When normality of paired differences was not rejected, bias was estimated as the mean difference, with 95% limits of agreement calculated as mean difference ± 1.96 SD. For AHI and left-side position, nonparametric estimates used the median difference and empirical 2.5th and 97.5th percentiles. Passing–Bablok regression and a two-way random-effects, single-measure intraclass correlation coefficient for absolute agreement [ICC(2,1)] provided complementary method-comparison analyses. Confidence intervals for Passing–Bablok intercepts and slopes were estimated using 10,000 participant-level bootstrap resamples, whereas ICC confidence intervals were calculated analytically. AHI severity-classification agreement was assessed using linearly weighted Cohen’s kappa. Sleep–wake agreement was evaluated using aligned, valid 30-s epochs, classified as sleep or wake. Epoch pairs were included only when both systems contained a corresponding sleep or wake run lasting ≥1.5 min in both systems. Overall percentage agreement and unweighted Cohen’s κ were calculated from pooled epoch counts, with 95% confidence intervals estimated using 10,000 participant-level bootstrap resamples. Statistical analyses were performed using MATLAB (R2026a; The MathWorks, Inc., Natick, MA, USA).

## 3. Results

A total of 26 children were enrolled, including 11 at Site A (Shamir Medical Center, Be’er Ya’akov, Israel) and 15 at Site B (Millennium Sleep Clinic, Be’er Sheva, Israel). Two participants withdrew consent, leaving 24 (92.3%) who completed simultaneous overnight recordings with both systems and were included in the final analysis. The median age of participants included in the final analysis was 10.5 years (25th–75th percentiles, 8.8–13.3; range, 6–17 years); eight participants (33.3%) were younger than 10 years, and 12 (50.0%) were female (Table 1). Participants presented with a range of underlying comorbidities, summarized in Supplementary Table S1.

**Figure 2:**
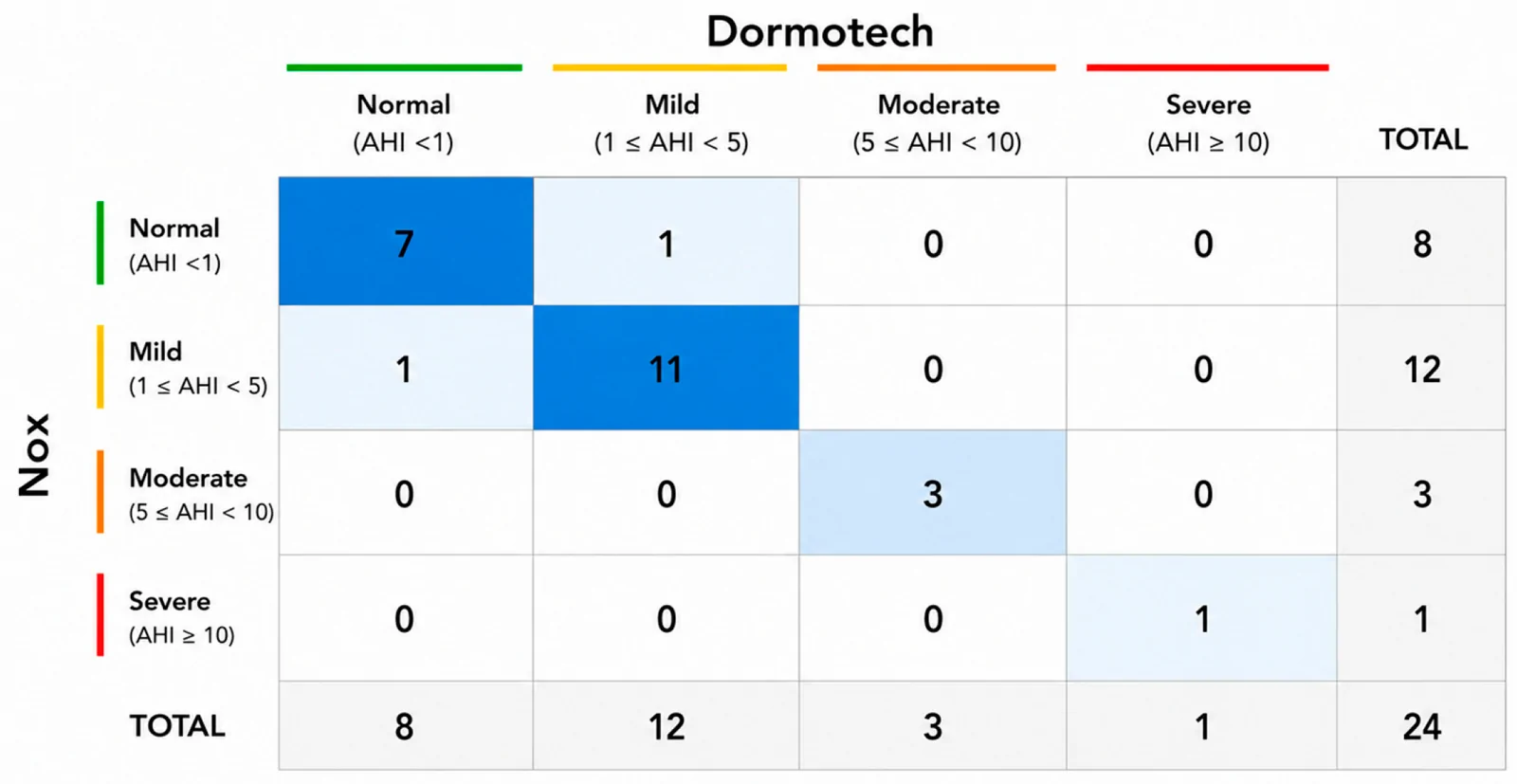
Agreement in pediatric AHI severity classification between Dormotech and reference polysomnography. Confusion matrix illustrating agreement in apnea–hypopnea index (AHI) severity classification between the investigational Dormotech system and the reference polysomnography (Nox A1) in 24 pediatric participants. Each cell represents the number of participants assigned to the corresponding severity category by both systems. The diagonal cells indicate concordant classifications, whereas off-diagonal cells represent discordant classifications. Marginal totals summarize the number of participants assigned to each severity category by each system. The color intensity of each cell reflects the number of participants, with darker shades indicating higher counts. Pediatric severity categories were defined according to AASM criteria: normal (AHI < 1 events/h), mild (1–<5 events/h), moderate (5–<10 events/h), and severe (≥10 events/h). Overall agreement between the two systems was 91.7% (22/24).

**Figure 3:**
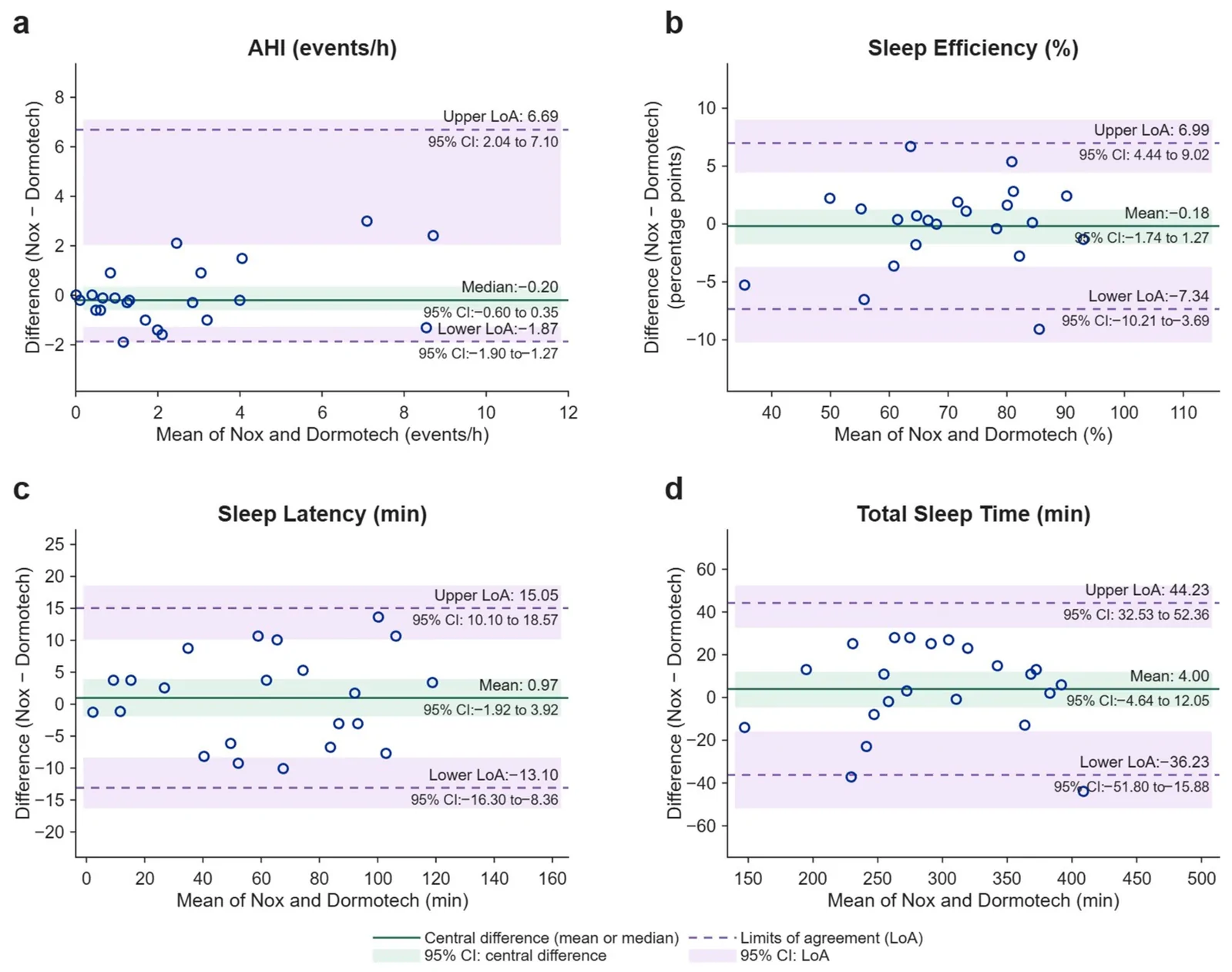
Bland–Altman agreement between Dormotech and reference polysomnography (Nox A1). Panels show (**a**) AHI (*n* = 24), (**b**) sleep efficiency (*n* = 22), (**c**) sleep latency (*n* = 22), and (**d**) total sleep time (*n* = 22). Differences (Nox − Dormotech) are plotted against the mean of the two measurements. For AHI, solid and dashed lines indicate the median difference and empirical 2.5th–97.5th percentile limits of agreement; for all other panels, they indicate the mean difference and mean ± 1.96 standard deviations. Shading indicates 95% percentile-bootstrap confidence intervals. One extreme AHI observation was retained in all calculations but is not displayed in panel (**a**).

Agreement in AHI severity classification was evaluated by independently assigning each participant to the standard pediatric severity categories using recordings from each system. As illustrated in the confusion matrix (Figure 2), the two systems agreed in 22 of 24 participants (91.7%). The two discordant cases (8.3%) occurred exclusively at the diagnostic boundary between normal and mild obstructive sleep apnea. One participant was classified as having mild OSA by Nox (AHI = 1.3 events/h) and normal breathing by Dormotech (AHI = 0.4 events/h), whereas the second participant was classified as normal by Nox (AHI = 0.2 events/h) and mild OSA by Dormotech (AHI = 2.1 events/h). AHI did not differ significantly between the systems (unadjusted *p* = 0.808; Table 2). Nonparametric Bland–Altman analysis showed a median paired difference of −0.2 events/h, with empirical limits of agreement of −1.9 to 6.7 events/h (Figure 3a). Agreement in severity classification was high (linearly weighted Cohen’s κ = 0.896), and the RMSE for AHI was 1.9 events/h.

No statistically significant differences were observed between the Dormotech and Nox systems for the reported global sleep–wake, respiratory, and body-position outcomes after adjustment for multiple comparisons (Table 2). Bland–Altman analyses showed small differences across the representative global sleep–wake measures shown in Figure 3b–d. Total sleep time showed the largest RMSE among these measures (20.4 min), with a mean difference of 4.0 min (Table 2).

Epoch-by-epoch sleep–wake agreement was assessed in 22 participants using 17,974 retained paired 30-s epochs. Pooled overall agreement was 85.44% (95% CI, 81.75–89.02%), with an unweighted Cohen’s κ of 0.666 (95% CI, 0.591–0.739).

**Figure 4:**
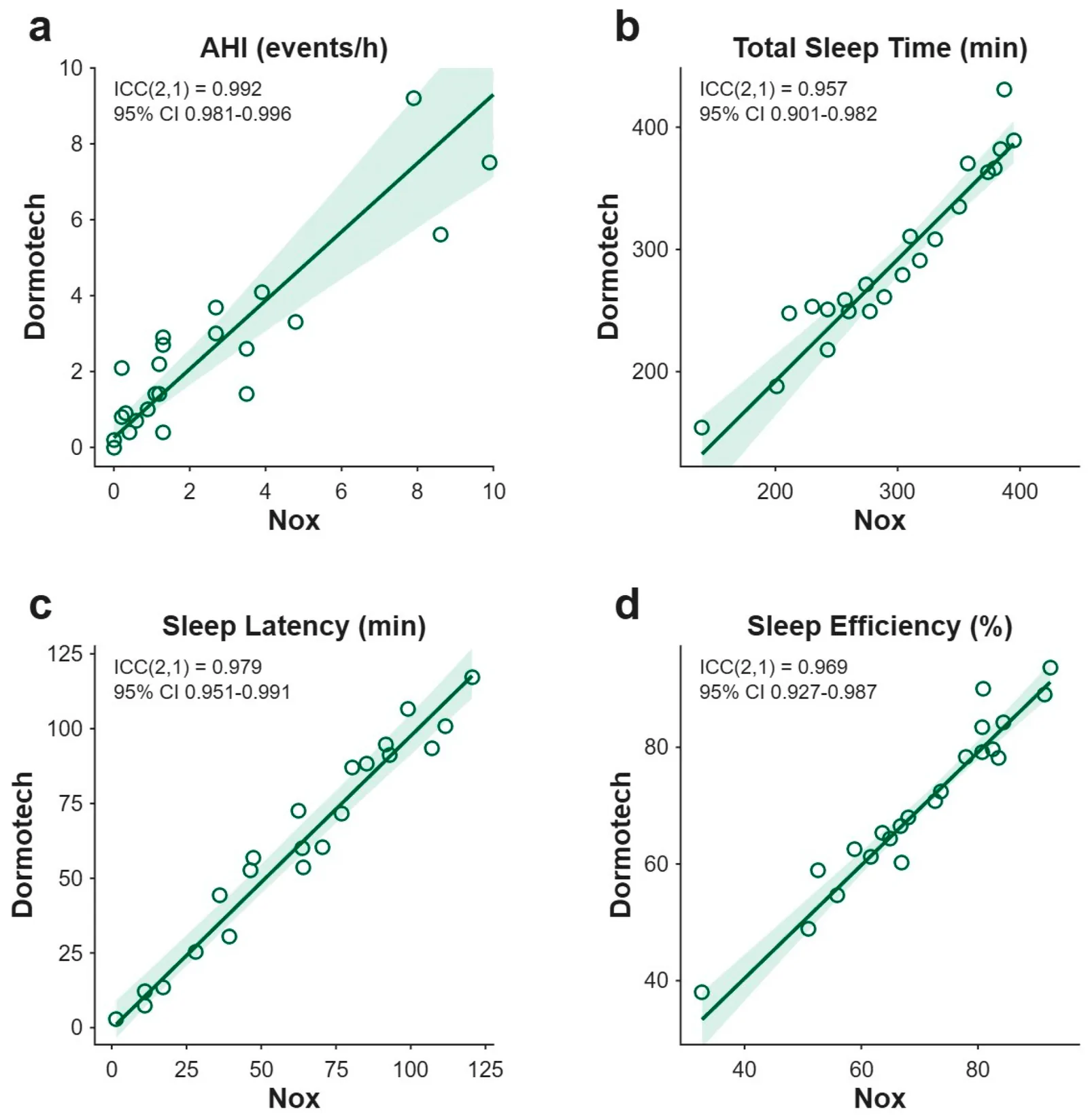
Passing-Bablok regression and intraclass correlation analysis comparing the Dormotech system with reference polysomnography (Nox A1). Scatter plots show paired measurements for (**a**) apnea–hypopnea index (AHI; *n* = 24), (**b**) total sleep time (*n* = 22), (**c**) sleep latency (*n* = 22), and (**d**) sleep efficiency (*n* = 22). Nox measurements are shown on the horizontal axis and Dormotech measurements on the vertical axis. Each point represents an individual participant. Solid lines indicate Passing-Bablok regression fits, and shaded regions indicate approximate 95% pointwise bootstrap confidence intervals for the fitted relationship. Intraclass correlation coefficients for absolute agreement [ICC(2,1)] and their 95% confidence intervals are displayed within each panel. All available finite paired observations were retained for each outcome; one participant with an extreme AHI value was included in all calculations but is not displayed in panel (**a**) for visual clarity.

Passing–Bablok regression showed that the 95% confidence intervals for the slopes included unity for AHI, total sleep time, sleep latency, and sleep efficiency, indicating no evidence of proportional bias between Nox and Dormotech. Absolute agreement was high across these measures, with ICC(2,1) estimates ranging from 0.957 to 0.992 (Figure 4). Detailed Passing–Bablok regression and ICC(2,1) estimates with 95% confidence intervals are provided in Supplementary Table S2. Device usability was evaluated using standardized questionnaires completed before and after the overnight study (Supplementary Tables S3 and S4). Median item ratings ranged from 4.0 to 5.0 before sleep and from 3.0 to 5.0 following the study. The proportion of ratings meeting the predefined acceptability threshold (score ≥ 3) ranged from 83% to 100% before sleep and from 71% to 100% following the study. No device-related adverse events were reported during the study.

## 4. Discussion

This study evaluated the investigational Dormotech Type 2 ambulatory sleep-monitoring system during simultaneous supervised laboratory recordings in children. The system showed agreement with reference PSG for respiratory indices, AHI severity classification, selected global sleep–wake measures, and epoch-by-epoch sleep–wake classification. These findings provide evidence of measurement agreement under controlled laboratory conditions and address an important unmet need in pediatric sleep medicine, where access to laboratory PSG remains limited and more accessible diagnostic approaches are needed [3,6]. The findings also extend previous validation of the Dormotech sleep platform in adults [10], adding evidence in a pediatric population with distinct physiological characteristics and diagnostic requirements.

The observed agreement is particularly relevant in pediatric sleep medicine, where respiratory event thresholds differ substantially from those used in adults and relatively small differences in respiratory event scoring or total sleep time may alter disease classification and clinical management [4]. Unlike respiratory-focused home sleep apnea tests, comprehensive Type 2 PSG systems can acquire both neurophysiological and respiratory signals relevant to pediatric sleep assessment. This distinction is especially important in children, in whom respiratory-event scoring and respiratory-index calculation are closely linked to neurophysiological sleep measures and accurate determination of total sleep time under AASM guidelines.

Agreement between the two systems extended well beyond respiratory event detection. No statistically significant differences were observed across secondary sleep, respiratory, and body-position outcomes after adjustment for multiple comparisons.

The concordance observed across these domains suggests that comprehensive Dormotech Type 2 PSG system preserves the broader physiological information routinely incorporated into pediatric PSG interpretation rather than providing only an estimate of respiratory disease severity.

Minor discrepancies in AHI severity classification were observed in two participants; however, both occurred at the diagnostic boundary separating normal findings from mild OSA. Because pediatric severity categories are defined using relatively narrow AHI thresholds, small absolute differences may result in categorical reclassification despite close numerical agreement. These classification differences likely reflect the inherent variability associated with respiratory event scoring near established pediatric clinical cutoffs. Such variability is well recognized in manual polysomnographic scoring, including repeated scoring by the same scorer, and is particularly pronounced when scoring hypopnea events [11,12]. The absence of systematic bias in AHI measurements and the excellent agreement in severity classification further support this interpretation that these isolated discrepancies do not represent systematic differences between the two recording systems.

Several limitations should be acknowledged. The relatively modest sample size limited statistical power for subgroup analyses according to age, disease severity, comorbidities, or specific sleep disorders. Although standard sleep staging was performed for each recording, this study was not designed to evaluate epoch-by-epoch agreement across individual sleep stages. Accordingly, the findings support agreement in global sleep–wake measures and sleep–wake classification, rather than validation of stage-specific scoring. All recordings were performed simultaneously in the sleep laboratory rather than independently in participants’ homes. This design was intentionally selected to isolate differences attributable to the recording systems while eliminating the confounding influence of physiological variability between nights. However, sleep recorded in the laboratory may not fully represent habitual sleep in children, whose sleep quality and architecture can be influenced by the unfamiliar environment [6,7]. In addition, the present study evaluated a single overnight recording and therefore did not account for the well-recognized night-to-night variability of pediatric sleep-disordered breathing. Previous studies have shown that AHI and OSA severity may vary across nights, particularly among children with mild disease or measurements near diagnostic thresholds [13,14,15]. Accordingly, disease severity may have differed if participants had been evaluated over multiple nights. Future studies incorporating multi-night recordings and evaluations in unsupervised settings may provide a more representative assessment of habitual sleep and respiratory physiology.

In conclusion, the Dormotech Type 2 PSG system, an ambulatory system designed for home sleep testing, demonstrated agreement with conventional laboratory PSG across respiratory events and selected global sleep–wake measures during simultaneous supervised laboratory recordings in children. Together with previous validation in adults, these findings extend the evidence base for comprehensive ambulatory Type 2 sleep monitoring to pediatric sleep medicine. As pediatric sleep care continues to move toward more accessible diagnostic pathways, technologies designed for home-based assessment, such as the Dormotech system, may provide a practical strategy for expanding access to sleep assessment while retaining neurophysiological and respiratory signals relevant to pediatric evaluation.

## Figures and Tables

**Table 1:** Demographic and baseline characteristics.

Characteristic	Value	%
**Description of enrolled participants (** ***n*** ** = 26)**		
Participants enrolled in the study	26	100.0
Participants who completed simultaneous recordings	24	92.3
Dropouts	2	7.7
**Description of participants included in the final analysis (** ***n*** ** = 24)**		
Age, years, median (25th–75th percentiles)	10.5 (8.8–13.3)	—
Age range, years	6–17	—
Female sex	12	50.0
Age < 10 years	8	33.3
BMI, kg/m^2^, median (25th–75th percentiles)	22.5 (15.9–33.3)	—
Participants contributing data to the final analysis	24	100.0
Reported device-related adverse events	0	0.0
**Sex by age group (** ***n*** ** = 24)**	**6–11 years**	**12–17 years**
Female	8	4
Male	7	5

Values are presented as median (25th–75th percentiles) unless otherwise stated. BMI, body mass index.

**Table 2:** Comparison of sleep parameters between Nox and Dormotech.

Parameter	Nox, Median (25th–75th Percentiles)	Dormotech, Median (25th–75th Percentiles)	*p* Value	RMSE	Bland–Altman Difference (LoA)
AHI, events/h	1.3 (0.6–3.6)	2.2 (0.9–3.4)	0.808	1.9	−0.2 (−1.9, 6.7)
ODI, events/h	0.9 (0.2–3.2)	1.2 (0.6–3.4)	0.798	1.2	−0.1 (−2.6, 2.4)
Snoring, %	17.5 (2.8–38.0)	19.9 (4.2–30.8)	0.381	5.3	2.0 (−7.8, 11.8)
Sleep latency, min	64.0 (37.0–90.0)	60.3 (34.1–90.5)	0.798	7.1	1.0 (−13.1, 15.0)
WASO, min	42.8 (21.3–79.3)	47.1 (23.2–83.4)	0.551	7.5	−2.1 (−16.6, 12.5)
Wake, %	28.4 (17.5–37.9)	30.7 (20.4–38.5)	0.381	3.2	−1.1 (−7.2, 5.0)
Total sleep time, min	296.5 (246.5–355.3)	275.0 (249.5–356.0)	0.680	20.4	4.0 (−36.2, 44.2)
Sleep efficiency, %	70.3 (62.1–80.9)	69.4 (61.5–79.6)	0.822	3.6	−0.2 (−7.3, 7.0)
Position, prone, %	2.9 (1.5–4.0)	2.9 (2.1–4.5)	0.600	1.2	−0.3 (−2.7, 2.1)
Position, supine, %	47.5 (23.9–73.5)	45.5 (25.2–69.8)	0.381	4.5	1.9 (−6.3, 10.1)
Position, left, %	14.2 (4.0–25.2)	14.5 (4.2–24.1)	0.798	3.1	0.0 (−3.2, 7.2)
Position, right, %	18.4 (4.9–35.7)	20.7 (4.0–34.5)	0.798	4.1	−0.3 (−8.5, 7.9)

Values are presented as median (25th–75th percentiles). Differences were calculated as Nox − Dormotech; differences and RMSE values for percentage outcomes are expressed in percentage points. The AHI *p* value is unadjusted; *p* values for the 11 secondary outcomes were adjusted using the Benjamini–Hochberg false-discovery-rate procedure. AHI, apnea–hypopnea index; ODI, oxygen desaturation index; WASO, wake after sleep onset; RMSE, root mean squared error; LoA, limits of agreement.

## Data Availability

Due to the sensitive nature of the clinical data, the datasets generated and analyzed during this study are not publicly available. Access may be granted by the corresponding author upon reasonable request and in accordance with institutional and ethical approvals.

## Supplementary Materials

The following supporting information can be downloaded at https://www.mdpi.com/article/10.3390/children13101432/s1: Table S1. Comorbidities reported among study participants; Table S2. Passing–Bablok regression and intraclass correlation analyses comparing Dormotech with reference polysomnography (Nox A1); Table S3. Usability Questionnaires; Table S4. Usability analysis of the Dormotech device based on subjective questionnaires.

## Abbreviations

The following abbreviations are used in this manuscript:

AASM	American Academy of Sleep Medicine
AHI	apnea–hypopnea index
BMI	body mass index
ECG	electrocardiogram
EEG	electroencephalogram
EMG	electromyogram
EOG	electrooculogram
FDA	Food and Drug Administration
FDR	false discovery rate
HST	home sleep testing
ICC	intraclass correlation coefficient
LoA	limits of agreement
N1, N2, N3	non-rapid eye movement sleep stages 1, 2, and 3
NREM	non-rapid eye movement
ODI	oxygen desaturation index
OSA	obstructive sleep apnea
PPG	photoplethysmography
PSG	polysomnography
REM	rapid eye movement
RMSE	root mean squared error
SD	standard deviation
SDB	sleep-disordered breathing
SpO_2_	peripheral oxygen saturation
TRT	total recording time
TST	total sleep time
WASO	wake after sleep onset
