# Supplementary material for: Advancing Sleep Testing for Children: In-Laboratory Validation of a Type 2 Sleep-Monitoring System Designed for Home-Based Pediatric Assessment
Source: Children (Basel). 2026 Oct 18;13(10):1432. doi: 10.3390/children13101432 (PMC13702878; doi:10.3390/children13101432)
Supplement: Supplementary file 1 [file children-13-01432-s001.zip › children-4526097-supplementary.pdf]

Supplementary Materials

Supplementary S1

Advancing Sleep Testing for Children: In-Laboratory Validation of a Type 2 Sleep-Monitoring System Designed for Home-Based Pediatric Assessment

Table S1. Comorbidities reported among study participants.

Comorbidity	Count	% of Total
Deviated nasal septum	2	8.3
Obesity / morbid obesity	3	12.5
Diabetes	2	8.3
ADHD	1	4.2
Cerebral palsy	1	4.2
Strep throat	1	4.2
Epilepsy	1	4.2
Lymphangioma	1	4.2
Asthma	2	8.3
PTSD	1	4.2
Autism spectrum disorder	1	4.2
Adenoid hypertrophy	1	4.2
Hodgkin lymphoma	1	4.2
None reported	9	37.5

ADHD, attention deficit hyperactivity disorder; PTSD, post-traumatic stress disorder.

Supplementary S2

This table summarizes the comorbid medical conditions reported by enrolled participants and their relative frequencies.

Table S2. Passing–Bablok regression and intraclass correlation analyses comparing Dormotech with reference polysomnography (Nox A1).

Parameter	Passing–Bablok intercept (95% CI)	Passing–Bablok slope (95% CI)	ICC(2,1) (95% CI)
AHI, events/h	0.257 (0.001 to 0.703)	0.904 (0.657 to 1.160)	0.992 (0.981 to 0.996)
ODI, events/h	0.316 (0.119 to 0.501)	0.920 (0.667 to 1.126)	0.998 (0.995 to 0.999)
Snoring duration, % of TST	0.965 (−0.112 to 2.974)	0.839 (0.764 to 0.978)	0.964 (0.912 to 0.985)
Sleep latency, min	−0.327 (−5.004 to 7.759)	0.979 (0.903 to 1.075)	0.979 (0.951 to 0.991)
Wake after sleep onset, min	2.066 (−4.701 to 6.912)	1.031 (0.919 to 1.108)	0.987 (0.970 to 0.995)
Wake, %	1.836 (−1.599 to 5.072)	0.983 (0.858 to 1.076)	0.974 (0.938 to 0.989)
Total sleep time, min	−6.728 (−70.305 to 46.842)	0.995 (0.831 to 1.175)	0.957 (0.901 to 0.982)
Sleep efficiency, %	1.639 (−7.328 to 10.210)	0.969 (0.851 to 1.096)	0.969 (0.927 to 0.987)
Position, prone, %	0.383 (−0.580 to 1.153)	1.022 (0.815 to 1.350)	0.972 (0.938 to 0.988)
Position, supine, %	0.829 (−2.852 to 3.856)	0.951 (0.892 to 1.013)	0.987 (0.967 to 0.995)
Position, left, %	0.000 (−0.828 to 1.277)	0.993 (0.906 to 1.053)	0.983 (0.962 to 0.993)
Position, right, %	−0.048 (−1.216 to 1.004)	0.950 (0.893 to 1.118)	0.970 (0.931 to 0.987)

Nox measurements were used as the reference (horizontal-axis) values and Dormotech measurements as the evaluated values. Passing–Bablok intercepts and slopes are reported with 95%

percentile-bootstrap confidence intervals. ICC(2,1) denotes a two-way random-effects, single-measurement intraclass correlation coefficient for absolute agreement; 95% confidence intervals were calculated analytically. Analyses used all available finite paired observations for each outcome without imputation. AHI, apnea–hypopnea index; CI, confidence interval; ICC, intraclass correlation coefficient; ODI, oxygen desaturation index; TST, total sleep time.

Supplementary S3

Device usability was assessed using pre-study (evening) and post-study (morning) questionnaires. Participants rated their agreement with each statement using a 5-point Likert scale (1 = strongly disagree, 2 = disagree, 3 = neutral, 4 = agree, 5 = strongly agree).

Table S3. Usability Questionnaires.

Pre-study questionnaire (evening / night).

Item	Statement
1	The head unit is comfortably positioned on the head.
2	The head unit is stably positioned on the head.
3	Using the head unit does not cause any discomfort.
4	No adaptation time is needed for the head unit to rest comfortably.
5	Wearing the head unit isn't causing any nasal congestion for me.
6	The body unit is comfortably positioned on the chest and abdomen.
7	The body unit is stably positioned on the chest and abdomen.
8	Using the body unit isn't causing any discomfort/pressure on the body.
9	No adaptation time is needed for the body unit to rest comfortably.
10	The investigator's instructions were clear to me.
11	I had an easy time following the instructions.
12	The user manual was clear.
13	The operation of the device was easy and clear.
14	The process of establishing the device's connectivity was easy and clear.
15	I was able to use the device without requiring assistance.
16	What rating would you assign to the device?

Post-study questionnaire (morning).

Item	Statement
1	The head unit was comfortably positioned on the head during the night.
2	The head unit was stably positioned on the head during the night.
3	Using the head unit didn't cause any discomfort.
4	No adaptation time was needed for the head unit to rest comfortably.
5	Wearing the head unit didn't cause any nasal congestion for me.
6	The body unit was comfortably positioned on the chest and abdomen during the night.
7	The body unit was stably positioned on the chest and abdomen during the night.
8	Using the body unit didn't cause any discomfort/pressure on the body.
9	No adaptation time was needed for the body unit to rest comfortably.
10	The investigator's instructions were clear to me.
11	The instructions were easy to follow, and I'm confident doing it on my own.
12	The user manual was clear.
13	The operation of the device was easy and clear.
14	The process of establishing the device's connectivity was easy and clear.
15	I was able to use the device without requiring assistance.
16	The device remained in place throughout the entire night.
17	The device didn't interrupt my sleep.
18	What rating would you assign to the device?

Supplementary S4

Table S4. Usability analysis of the Dormotech device based on subjective questionnaires.

Question	Morning / after study		Night / before study	
	Median (25th–75th percentiles)	Responses ≥3, %	Median (25th–75th percentiles)	Responses ≥3, %
1. Head unit comfort	3.5 (3–4)	83	4 (3–5)	92
2. Head unit stability	4 (3–4)	96	5 (4–5)	96
3. Head unit, no discomfort	3.5 (2–4)	75	4 (3–4)	83
4. Head unit adaptation	3 (2–4)	71	4 (3–5)	88
5. No nasal congestion	4 (4–5)	88	5 (3–5)	83
6. Body unit comfort	4 (4–5)	83	5 (4–5)	100
7. Body unit stability	4 (4–5)	92	5 (5–5)	100
8. Body unit, no discomfort	4 (3–5)	79	5 (4–5)	100
9. Body unit adaptation	4 (4–5)	92	5 (4–5)	100
10. Instructions were clear	5 (4–5)	96	5 (4–5)	100
11. Instructions easy to follow	3.5 (2–4)	71	4 (4–4)	96
12. User manual was clear	4 (3–4)	100	4 (4–4)	100
13. Operation easy and clear	3 (3–4)	96	4 (4–4)	100
14. Connectivity easy and clear	4 (3–4)	92	4 (4–4)	100
15. Usability without assistance	3 (2–4)	71	4 (3–4)	96
16. Night: overall device rating; morning: device remained in place	4 (4–5)	92	4 (4–5)	96
17. Device did not interrupt sleep	3.5 (2–4)	75	—	—
18. Overall rating of device	4 (3–4)	96	—	—

Responses were recorded on a 5-point Likert scale (1 = strongly disagree; 5 = strongly agree) and are presented as median (25th–75th percentiles). Responses rated ≥3 represent the percentage of participants reporting a neutral or more favorable response. n = 24 for all items except morning Question 13 (n = 23 because of one missing response).
